# Psychometric evaluation of the Thai version of the Early Childhood Oral Health Impact Scale (Th-ECOHIS): a cross sectional validation study

**DOI:** 10.1186/s12903-020-01332-y

**Published:** 2021-02-11

**Authors:** Pattarawadee Leelataweewud, Varangkanar Jirarattanasopha, Chantana Ungchusak, Warangkana Vejvithee

**Affiliations:** 1 Department of Pediatric Dentistry, Faculty of Dentistry, Mahidol University, No. 6, Yothi Road, Ratchathewi District, Bangkok, 10400 Thailand; 2 Bureau of Dental Health, Department of Health, Ministry of Public Health, No. 88/22, Tiwanond Road, Nonthaburi, 11000 Thailand

**Keywords:** Oral health-related quality of life, Early childhood Oral health impact scale, Early childhood caries, Preschool children, Validation

## Abstract

**Background:**

Early childhood caries (ECC) is prevalent in Thailand, but no appropriate tool has been available to measure its impact on children’s quality of life. This study translated the Early Childhood Oral Health Impact Scale (ECOHIS), a widely used proxy-reported questionnaire developed in the United States for measuring the oral health-related quality of life of preschool children and their families, into Thai (Th-ECOHIS). The scale’s psychometric properties were investigated in Thai caregivers and their children.

**Methods:**

Cultural adaptation for the scale development within the Thai context was processed using forward–backward translation by experts. A face and content validation was conducted among 20 Thai caregivers to attain the final Th-ECOHIS. Psychometric testing was done on 3-year-old child-caregiver pairs in Bangkok using the interviewer-administered mode. Children’s oral health was determined by caries experience (decayed, missing and filled primary teeth, dmft) and treatment need. The caregivers answered the Th-ECOHIS and global questions regarding their perception of the children’s oral health. Across-items reliability was assessed by internal consistency using the Cronbach’s alpha coefficient. Test-retest reliability was managed at a 2-week interval in 10% of the sample using the intraclass correlation coefficient calculated by two-way analysis of variance. The discriminant validity was tested by the relationship between the severity of dental caries, treatment need and Th-ECOHIS scores, using the Kruskal-Wallis test.

**Results:**

A total of 214 child-caregiver pairs participated. Twenty-two percent had ECC (dmft 1–3) and 17.3% had severe ECC (dmft 4 or higher) with mean (SD) dmft 1.63 (2.92). All items in the original ECOHIS were retained in the Thai version. The test-retest reliability of Th-ECOHIS was 0.87; internal consistency was 0.85; the total Th-ECOHIS scores were significantly correlated with the global rating of oral health question (*r* = 0.604). Th-ECOHIS scores in both child and family impact sections and the total were significantly associated with the severity of caries (*p* <  0.001) and treatment need (*p* <  0.001).

**Conclusions:**

Th-ECOHIS demonstrated good reliability and validity. It could be used on caregivers to assess the impacts of ECC on quality of life of Thai pre-school children and compared to other countries.

## Background

Early childhood caries (ECC) has been recognized by health care professionals as one of the major health problems in young children around the world. The severity of the problem is commonly indicated by clinical indicators such as the prevalence and number of decayed, missing, and filled teeth (dmft). According to a recent systematic review, worldwide prevalence of ECC ranged from 23 to 90% [1]. Seventy percent of included countries reported a prevalence of ECC in 5-year-old children higher than 50%.

A variety of attempts have been made to demonstrate the negative impacts of ECC. Objective parameters, such as a child’s body weight and height, restricted growth, and future dentition problems, have been shown as evidence of the impact [2–6]. However, these objective parameters have had little influence in getting caretakers of ECC-susceptible children to give priority to preventive practices. Subjective assessments related to pain, school performance, and restriction of family function have also later demonstrated the impact of ECC [7, 8].

Preschool children could be subjected to oral discomfort from various origins: teething pain [9], traumatic dental injury [10], oral ulceration [11] and caries-related conditions [12, 13]; most have been reported to impair children’s quality of life. The measurement of oral health-related quality of life (OHRQoL) of children is recognized as the integration of many aspects including physical, psychological, social, and functional dimensions [7, 8, 14–16]. These aspects cannot be assessed easily by objective indicators. Instead, comprehensive multidimensional and subjective evaluations are needed to capture the effects of ECC on these dimensions. Subjective assessment of young children is difficult due to their level of cognitive function [17, 18], yet it is necessary. To evaluate a young child’s quality of life, family and caretakers are vital because they are able to perceive the child’s needs and are aware of problems in fulfilling those needs. They can also speak up for young children who may not be able to do so for themselves [7]. Furthermore, they may be indirectly affected by a child’s oral health problems [7].

The Early Childhood Oral Health Impact Scale (ECOHIS) was developed in the United States by Pahel and colleagues and published in 2007 [8]. It specifically measures the impact of ECC on a preschool child’s quality of life. It was designed to assess a child’s oral function, social function, and psychological performance, as well as the indirect effects of ECC on family distress and function. To overcome the limitations of a young child’s ability to express thoughts and respond to questions, the ECOHIS focuses on the perception of parents or main caregivers. It has been translated into several languages and cross-culturally adapted for many countries worldwide, including France, China (Hong Kong), Turkey, Brazil, Venezuela, Lithuania, Iran, Malaysia, Saudi Arabia, Chile, Germany and Madagascar [19–31]. All translations have exhibited a high degree of reliability and validity [19–31]. The ECOHIS has become a widely used tool for showing the impact of ECC on a child’s quality of life.

Dental caries in preschool children has been a health problem in Thailand for many years [32, 33]. Several strategies have been implemented to lower the prevalence of ECC, but two consecutive National Oral Health Surveys (NOHS) at a 5-year interval revealed that the prevalence remained high [32, 33]. Raising awareness of the impact of ECC on children in terms of parents’ perceptions would be essential to help design more effective and suitable preventive strategies and interventions. Studies from many parts of the world have used ECOHIS as a tool and confirmed the negative impact of ECC on quality of life [34]. However, not one tool in the Thai language was available to assess OHRQoL in young children. A recent systematic review of the advantages of available instruments for OHRQoL measurement also found that ECOHIS was the most robust tool suitable for preschool children [35]. Therefore, this study aimed to translate the ECOHIS into the Thai language and validate the Thai version of the ECOHIS (Th-ECOHIS).

## Methods

**Fig. 1:**
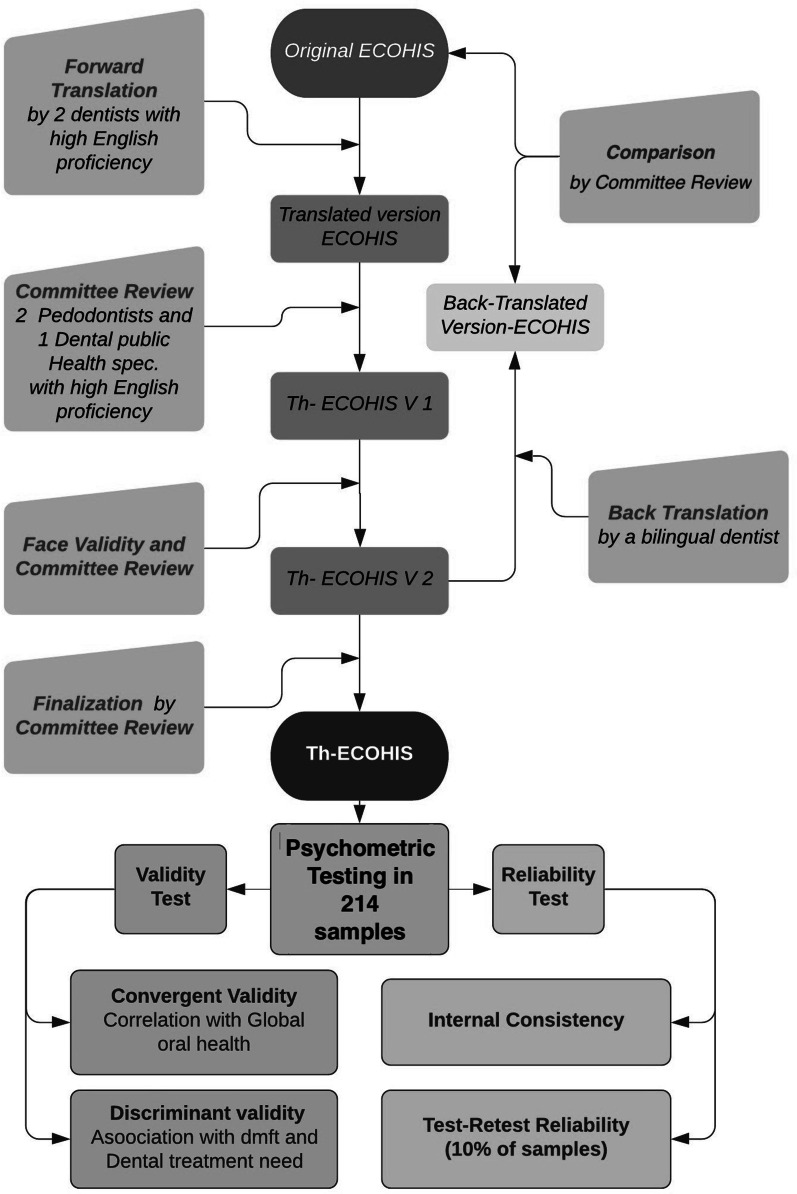
Translation, face and content validation process, and psychometric investigation

The study protocol was approved by the Ethical Review Committee for Research in Human Subjects, Thailand Ministry of Public Health. Written informed consent was obtained from primary caregivers for their participation in the study. The process of developing a Thai version of the ECOHIS consisted of two main phases: 1) translation, face and content validation, 2) psychometric testing (Fig. 1).

### Translation, face and content validation phase

The translation and cross-cultural adaptation process of the original ECOHIS [8] into the Thai version followed international standards for the development of new linguistic versions [36, 37]. The original English version of ECOHIS was translated into Thai by two dentists with high English proficiency. Content validation to ensure equivalency to the original version was conducted by a committee of English-proficient Thai specialists in pediatric dentistry and public health who were familiar with a quality-of-life assessment. Each questionnaire item was reviewed and discussed in terms of its concept being consistent with the original version until a first draft of the Thai version was reached.

Face validation of the first draft of Th-ECOHIS was conducted in a convenience sample of 20 Thai primary caregivers of kindergarten children in a Bangkok suburb. After a questionnaire interview, all participants were invited to discuss the clarity and comprehensibility of each questionnaire item, the logic and relevance of the order of the items, and the understandability of the rating scale for answers. The committee did another review to improve each item using all comments from the face validation to produce the second draft of Th-ECOHIS.

The second draft was then blind-backward translated by a bilingual dentist who was a native speaker of both Thai and English. The committee compared the back-translated ECOHIS with the original version. The differences found between the two versions were minor; the translated version was consistent with the original version, item by item. The working group reconsidered, reviewed all the details and finalized a Thai version of ECOHIS.

### Psychometric testing phase

In order to evaluate reliability and validity of the Th-ECOHIS, a psychometric testing phase was conducted.

#### Sampling

The sample size for psychometric testing was calculated according to internal consistency for Cronbach’s alpha using Bonnett’s formula [38] for 13 question items. The required level and planning value for Cronbach’s alpha were set at 0.70, and 0.80, respectively, based on a previous study [26] against a two-sided alternative at α = 0.05 with power of 90%. With a 20% dropout rate, at least 176 subjects were required for the study. The sample size for test-retest reliability was set using an estimated sample size table calculated from a well-received formula proposed by Walter et al. [39]. The acceptable reliability and expected reliability were set at 0.7 and 0.9, respectively, at α = 0.05 and β = 0.2. The minimum required sample size was 19. With a 10% dropout rate, 21 subjects, approximately 10% of participants, were invited for test-retest reliability.

This phase was conducted in a subset of Bangkok samples of the 7th Thailand NOHS [33] where a systematic sampling design was deployed using districts based on urban and suburban zoning and subsequently, subdistricts as a sampling unit. Our study randomly selected one subdistrict from the designated subdistricts. The total sample of child-caregiver pairs in three randomly selected kindergartens in the specified subdistrict were simultaneously invited into this study along with the 7th Thailand NOHS recruitment. To be included, the child had to be 36 to 48 months of age and able to cooperate for a dental caries examination. The proxy respondent had to be a parent or main caregiver who took primary responsibility for the child, and literate in the Thai language. The only exclusion criterion was inability to complete the interview session. The schools were asked to arrange participant interview sessions around the designated time for student pick-up.

#### Questionnaire

The Th-ECOHIS questionnaire had two main parts containing a total of 13 items, as did the original. Nine items measured the impact of ECC on a child in four aspects: symptoms (1 item), function (4 items), emotional well-being (2 items), and self-image and social interaction (2 items). The other four items evaluated the impact on the family in two aspects: parental distress (2 items) and family function (2 items). Questions required parents or caregivers to recall how often they had observed their child and family encountering situations involving their child’s oral health since the child was born. Responses were formatted in a simple five-point Likert-type scale, with answers ranging from Never (0) to Very Often (4); a response of Don’t Know was an alternative response.

To evaluate the convergent validity of the Th-ECOHIS questionnaire, one global oral health rating question was added to the questionnaire: “In general, how would you rate the dental health of your child?” The response codes were 1 = excellent, 2 = very good, 3 = good, 4 = fair, and 5 = poor. Our study hypothesized that children whose general oral health was rated as poor would get high scores on both parts of the Th-ECOHIS.

#### Data collection

The main caregivers were interviewed using the structured Th-ECOHIS questionnaire by one interviewer and asked to provide demographic information. To avoid bias, the interviewer read the questions to the participants without explaining any of the questions or elaborating on any of the responses. For the rating scale, participants were shown a series of scale cards and asked to choose their response from the ones on the cards; their responses were then recorded by the interviewer. After 2 weeks, 10% of the caregivers were invited to a second interview at which they were asked the same questions.

The child’s weight and height were measured as general health indicators. The oral health status of each child was indicated by the dental caries experience index and dental treatment need adopted from the 7th Thailand NOHS [33]. It was conducted by trained and calibrated examiners based on the World Health Organization basic criteria for the visual assessment of dental caries. The dental caries status of each child was classified into three groups by the number of decayed, missing, and filled teeth (dmft index): caries free; ECC (dmft 1 to 3); and severe ECC (dmft 4 or higher). The treatment needs were classified into three categories: no treatment need, need for restoration, and need for pulp treatment and / or extraction.

#### Data analyses

The Th-ECOHIS scores were calculated as a simple sum of the response codes for the child impact section, family impact section, and overall questionnaire. The Don’t Know response was recoded as a missing value. Our study managed the missing response by adapting the method proposed in the original version [8]. For subjects with up to two missing responses on the child impact section or one missing response on the family impact section, a score for the missing item was imputed as a median score of the remaining items for that section. Participants with more than two missing child items and one missing family item would be excluded from the analysis. The psychometric testing of the Th-ECOHIS was analyzed by assessing its internal consistency and test–retest reliability, as well as its convergent and discriminant validity.

The child impact section, family impact section, and overall questionnaire were assessed for internal reliability, using Cronbach’s alpha coefficient. The test–retest reliability was assessed using the intraclass correlation coefficient (ICC) calculated by a two-way analysis of variance. The convergent validity was determined by computing the Spearman’s rank-order correlations of the responses to the global question and each of the Th-ECOHIS sections and overall scores. The discriminant validity was tested by the Kruskal–Wallis test to compare the scores of the child impact section, family impact section, and overall questionnaire among children with different severities of caries experience and treatment need. The SPSS statistical package version 17 was used for data analysis. The level of statistical significance is set at a *p*-value < 0.05.

## Results

### Translation, face and content validation phase

All 13 items in the original ECOHIS were translated into Thai, incorporating cross-cultural concerns with an awareness of the lexical gaps between Thai and English. The translation was processed based on conceptual equivalence rather than linguistic/literal equivalence. Two translated copies from the blind parallel translation were compared and merged. The committee review for content equivalence adjusted the word *difficulty* which has many variations in Thai when used in different contexts; for instance, the English phrase “difficulty pronouncing” was translated as “speech sound errors” in Thai, and the English phrase “difficulty eating” was translated as “pain when chewing” in Thai.

The 20 participating primary caregivers in face validation process were 16 mothers, 2 fathers and 2 relatives of kindergarten children with a mean age (SD) of 42.7 (7.4) months. All questions appeared to be well understood and none received a “Don’t know” response. Comments from the participants and interviewers during face validation suggested question sequence changes to smooth the transition between questions for better flow and to promote recalls.

All 13 questions from the original ECOHIS were kept in the Thai version. The sequence of questions was adjusted. The psychological section, oral problems causing *irritability or frustration and sleep problem,* was moved to after the child symptom section because the section concerned child’s suffering that could be most noticeable. The functional section, the question involving *“missing school”* was moved to be the first in the section because school attendance is given priority in the Thai culture and would be the most memorable and easiest to recall.

### Psychometric testing phase

A total of 240 child-caregiver pairs were recruited, and 214 pairs completed the data for analysis. Sixteen pairs were excluded because their caregivers had limited time to complete the Th-ECOHIS questionnaire. Of the 214 remaining caregivers, 65.4% (*n* = 140) were mothers, 75.2% (*n* = 161) were from families with an average income, and 97.6% were Buddhists. All children were healthy, most had weight (88.3%) and height (92%) appropriate to their age (2SD) [40]. The mean (SD) age of the children was 42.93 (2.86) months, ranging from 36 to 48 months. Of the 214 children, 50% were boys, 60.7% were caries-free and 17.3% had severe ECC (dmft 4 or higher). The mean (SD) dmft score was 1.63 (2.92). The demographic information of the children and their proxy respondents is shown in Table 1.

**Table 1:** Demographic data of children and proxy respondents

	Total	Caries free	ECC (dmft 1–3)	Severe (dmft ≥4)	*p-*value*
Number of child-caregiver pairs, n (%)	214 (100%)	130 (60.7%)	47 (22.0%)	37 (17.3%)	
*Child demographic data*					
Gender					0.389
Male	107 (50.0)	64 (49.2)	21 (44.7)	22 (59.5)	
Female	107 (50.0)	66 (50.8)	26 (55.3)	15 (40.5)	
Mean age (month)(SD)	42.93 (2.86)	42.98 (2.84)	42.94 (2.93)	42.70 (2.92)	0.870
Min - Max	36–48	38–48	38–48	37–48	
*Proxy demographic data*					
Relationship to the child					
Mother	140 (65.4)	88 (67.0)	30 (63.8)	22 (59.5)	0.449
Father	47 (22.0)	24 (18.5)	11 (23.4)	12 (32.4)	
Others	27 (12.6)	18 (13.8)	6 (12.8)	3 (8.1)	
Monthly family income^a^					0.043
Low income (< 20,000 bath)	38 (17.7)	19 (14.6)	15 (31.9)	4 (10.8)	
Average income (20,000–50,000 bath)	161 (75.2)	100 (76.9)	31 (66.0)	30 (81.1)	
High income (> 50,000 bath)	15 (7.01)	11 (8.5)	1 (2.1)	3 (8.1)	

*Comparison among caries free, ECC and severe ECC group

^a^According to The National Statistics Office household income survey (by type of occupation) in Bangkok 2011 [41]

All of the proxy respondents completed the questionnaires with no blank items and no more than one “Don’t know” response item for each child. The distribution of the proxy responses to the Th-ECOHIS items related to a child’s oral health problems is shown in Table 2, and the descriptive distribution by section is summarized in Table 3. The maximum scores of the Th-ECOHIS were 27 of 36 in the child impact section and 12 of 16 in the family impact section. The ceiling effect was negligible for each item and for the total score. Two respondents answered “Don’t know” to the item “child avoided talking,” and two respondents answered "Don’t Know" to the item “family member felt guilty.”

**Table 2:**
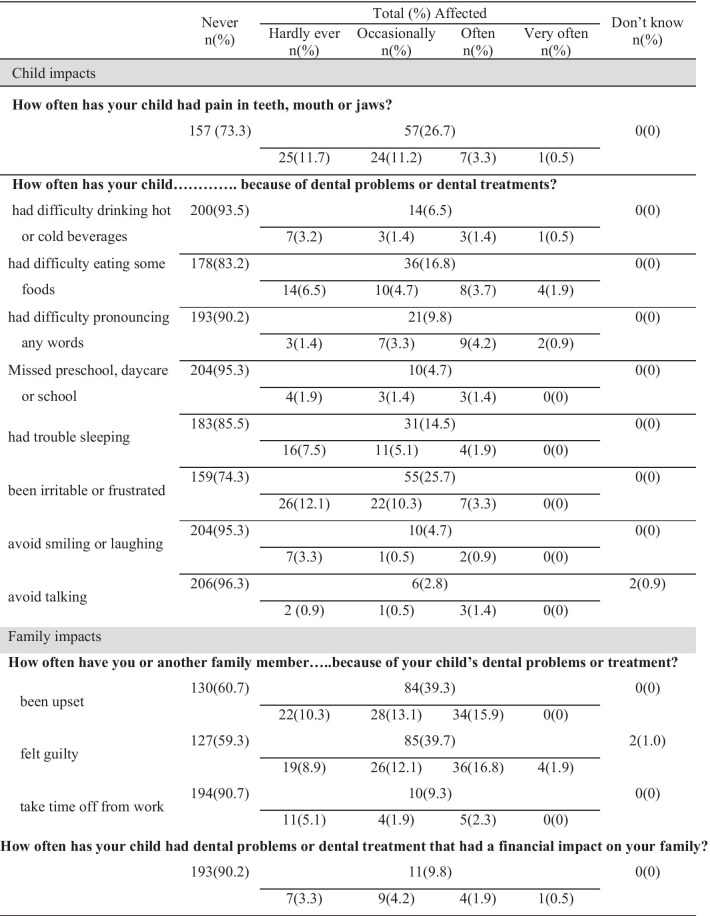
Distribution of the proxy responses to the Th-ECOHIS items related to child’s oral health problems

**Table 3:** Descriptive distribution of the Th- ECOHIS by sections

Impacts	Number of items	Possible range	Range	Floor effect (%score = 0)	Affected response (%)	Mean (SD)	Median IQR
Child impact section	9	0–36	0–27	63.1	36.9	2.05 (3.94)	0 (3)
Child symptoms	1	0–4	0–4	73.3	26.7	0.46 (0.853)	0 (1)
Child function	4	0–16	0–12	77.1	22.9	0.80 (1.926)	0 (0)
Child psychology	2	0–8	0–6	72.0	28.0	0.66 (1.307)	0 (1)
Self-image and social interaction	2	0–8	0–6	93.9	6.1	0.13 (0.693)	0 (0)
Family impact section	4	0–16	0–12	53.7	46.3	2.10 (2.783)	0 (4)
Parental distress	2	0–8	0–6	55.1	44.9	1.75 (2.264)	0 (4)
Family function	2	0–8	0–6	85.5	14.5	0.35 (1.018)	0 (0)
All sections	13	0–52	0–39	44.9	55.1	4.15 (5.980)	2 (6)

## Discussion

The original ECOHIS aimed to assess the impact of both oral health problems and related treatment experiences on the quality of life of preschool children and their families [8]. This study is the first attempt to deploy a valid cross-cultural instrument that has been used effectively in many countries worldwide to measure OHRQoL of preschool children in Thailand. ECOHIS focuses on the impact of ECC, which has been a major health problem in Thai preschoolers. The most recent NOHS in Thailand in 2017 revealed that the prevalence of ECC was 52.9% in 3-year-olds and 75.6% in 5-year-olds [32]. An assessment of the impact of these oral health problems on children’s quality of life has never been conducted. The ECOHIS has been adapted for use in some Asian populations with a similar ECC situation as in Thailand [22, 25]. The Thai version of ECOHIS was developed from the original version following the standard cross-cultural adaption process [36, 37]. All 13 items of the original ECOHIS were retained, and thus the Th-ECOHIS could be employed in cross-national comparisons. However, some minor revisions were made to be more applicable for the Thai context.

The original version of the ECOHIS was developed as a self-completed questionnaire by proxy [8]. Although the Th-ECOHIS kept the original context, it was interviewer-administered in this study. The interview approach was considered suitable for the studied population because it is a personal approach, ensures full return rates, lessens the possibility of missing data, and is non-literacy dependent. Although the literacy rate of working-age adults in Thailand is about 82%, the literacy rates of females and older persons are lower, at 78 and 52%, respectively [42]. One third of Thai adults aged 25 to 50 years are not fond of reading [42]. This was confirmed during the face validation process, when the mode of administration was discussed; the participants preferred the interviewing mode because they felt it would help them focus on the questions and process them better. Both self- and interviewer-administered Brazilian versions of ECOHIS were shown to have similar levels of reliability and validity and psychometric findings [30]. Studies using other OHRQoL instruments have demonstrated that the mode of administration does not affect the performance of the measure [43, 44]. However, self-administration has more advantages in terms of lower cost, the preservation of participants’ anonymity and autonomy, avoidance of interviewer bias, and availability for a large number of samples. The Th-ECOHIS questionnaire in self-completed mode should be tested in future studies.

Most of the caregivers who participated in this study were able to rate their children’s and family’s experience as asked in the Th-ECOHIS. The fact that there were only four responses of “Don’t Know” (1%) implies that only few caregivers had underestimated the effects of ECC. There were no blank responses in our study because the interview-administration mode encouraged respondents to answer each question. Approximately one half of caregivers reported at least one impact on either their children or families, or both (Th-ECOHIS score > 0). The ceiling Th-ECOHIS score of each section was not detected, and this was consistent with other validation studies [8, 21, 22, 24, 29]. Regarding the floor effect, our study showed a higher rate in the child impact section than did the original study [8]; it is possibly contributed from the lower percentage of affected children in this study. The participants recruited in this study were limited to 3-year-olds, whereas the original study included samples of 5-year-olds [8]. The younger age group could have been less affected by ECC, with a lower level of severity causing fewer symptoms and less impairment than the older age group could have. However, the three most common problems reported in the child impact section, pain, irritation and frustration, and eating difficulty, were similar to those reported in the original version [8] and in other countries with different cultures [22, 26, 30]. Apparently, children’s physical complaints, psychological effects and limited routine function were more easily detected by parents than were children’s social effects. In the family impact section, as in some other studies [8, 22, 26, 30], the most frequent feelings of caregivers were guilt and being upset. Although dental insurance is not available in Thailand, most simple dental services are provided free of charge for children younger than 12 years; only 10% of caregivers reported experiencing a financial impact on their family.

The reliability assessment of the Th-ECOHIS demonstrated an excellent result similar to the original version and previous studies. The test–retest analysis that was conducted over a period of 2 weeks revealed good agreement (0.87), indicating that the Th-ECOHIS questionnaire was able to yield stability of the scores when administered at two different times. The ICC value was similar to that reported for the original English, German, and Farsi versions of the ECOHIS (0.84, 0.81, and 0.82, respectively) [8, 19, 21].

Regarding the consistency of results across items within questionnaire, the overall Cronbach’s alpha value was 0.85, demonstrating good internal reliability of the Th-ECOHIS. This value was slightly lower than that reported for the original English [8] and German versions [19] but was within the same range reported for the Arabic, Brazilian, Lithuanian, and Malay versions [21, 22, 24, 30]. Comparing the internal consistency of the child impact section and the family impact section, the Cronbach’s alpha coefficient in the family impact section (0.71) was lower than that in the child impact section (0.84). This was similar to previous studies in which the internal consistency in the child impact section and the family impact section ranging from 0.74 to 0.92 and 0.59 to 0.85, respectively [8, 19–31]. The smaller number of items in the family impact section in Th-ECOHIS might have been one of the factors influencing this lower consistency.

To assess validity of the Th-ECOHIS, convergent and discriminant analyses were conducted. Both analyses showed that the Th-ECOHIS had good validity. Our study used the global measure of oral health to assess the convergent validity of the Th-ECOHIS. This measure is commonly used as a subjective indicator and has been shown to highly correlate with the clinical oral health status [45]. The Th-ECOHIS showed a moderate correlation with the global measure of oral health. The correlation of our finding was higher than in the original version [8] and was comparable to the Turkish and Brazilian versions [29, 30]. This finding showed that parents who perceived their children’s oral health as poor tended to have a higher ECOHIS score.

In the discriminant validity analysis, our study compared the Th-ECOHIS scores among children with different caries status and treatment needs. The results clearly suggested the Th-ECOHIS score could be a valid indicator of compromised quality of life of children for different severity of caries and treatment needs. Children with a more severe caries category and treatment need had higher scores in both total and sub-sections. Other studies found that the treatment needs showing an effect on children’s quality of life were those related to pulpal involvement and pain [31, 46]; however, in our study, even non-pulpal-involved treatment need showed an effect on children’s OHRQoL. This also implied that parental responses are reliable for assessment of their child’s quality of life based on the child’s oral health. It was also noted that the ECOHIS scores were reported for the child and family impact sections even in caries-free children. ECOHIS might be able to detect other oral problems not limited to dental caries.

Most studies of translated versions of the ECOHIS focused on a mixed-age group of preschoolers [19–31], while the original focused on only 5-year-olds [8]. Our study concentrated on 3-year-olds, the youngest group included in the NOHS and also targeted for early intervention in health policy. Two recent consecutive Thai NOHSs have shown high ECC prevalence in this age group [25, 26], approximately 53% with mean dmft of 2.8 in the country and 49.5% with dmft of 2.6 in Bangkok. Assessment of OHRQoL in this age group was scarce and deserves more attention. The ECOHIS score could be a simple subjective indicator of their quality of life. However, validation in the younger age group with relatively lower oral problems might raise some concerns regarding possible less relevant question items or poor responses by the proxies. In our study, full response from caregivers was achieved through the design of the interview-administered questionnaire; thus, the age group of the children should not have affected the validity and reliability of the instrument. On the other hand, it might affect the magnitude of the impact of oral health problems. The ECOHIS was proved valid for this particular age group.

It should be noted that the study was carried out in a specific group of children in the capital city from homogeneous socioeconomic status, middle-income families. In addition, we limited the socio-demographic characteristics of caregivers with respect to their relationship to the child and monthly income per household; age and level of education of caregivers were not collected. Further studies in different age groups and with a diversity of background would help strengthen our results. Future studies would also find it noteworthy to include more general health parameters such as perceived general health, general health behaviors, children’s weight, and height for convergent validity testing of this scale. However, those parameters at this young age with limited ECC severity might not show a correlation with or an impact on OHRQoL as in this study, all child samples were healthy with normal weight and height, and no any growth alteration was detected.

It could also be a limitation in this study that classical test theory (CTT) was used for psychometric testing of the scale, similar to what has been used in many previous studies [19–31]. All versions have kept all items as in the original. This would be useful in across-countries comparison of children’s OHRQoL measured by different versions of the ECOHIS. The CTT treats all questions equally, which contributes to simplicity in analysis and familiarity in dentistry. However, it would be useful to further analyze the translated version using item response theory (IRT) such as Rasch analysis [47, 48]. This would help detect any misfit items considered for modification or shortening the scale to customize it specifically for the Thai population.

This patient-based outcome measure will be a very useful parameter in demonstrating compromised quality of life in variety groups of preschool children. It could be incorporated into the National Oral Health Survey. Illustrating the deleterious effects of ECC would raise awareness in parents and families of the need for caries prevention. It could help improve communication among dentists, patients and policy makers. Future studies on responsiveness of the Th-ECOHIS would be necessary prior to applying the tool as a metric parameter to evaluate various intervention programs for ECC-affected children.

## Conclusion

The overall psychometric evaluation of the Th-ECOHIS was demonstrated to be valid and reliable for evaluating the impact of oral health problems on the quality of life of Thai preschool children and their families. The Th-ECOHIS can be used worldwide in cross-cultural comparisons with other language-translated ECOHIS studies.

## Data Availability

The datasets used and analyzed during the current study are available from the corresponding author upon reasonable request.

## Abbreviations

ECC: Early childhood caries
ECOHIS: Early Childhood Oral Health Impact Scale
Th-ECOHIS: Thailand version of the Early Childhood Oral Health Impact Scale
dmft: Decayed, missing and filled teeth
SD: Standard deviation
OHRQoL: Oral health-related quality of life
NHOS: National Oral Health Survey
ICC: Intraclass correlation coefficient

## References

[1] Chen KJ, Gao SS, Duangthip D, Lo ECM, Chu CH (2019). Prevalence of early childhood caries among 5-year-old children: a systematic review. J Investig Clin Dent.

[2] Shen A, Bernabé E, Sabbah W (2019). The bidirectional relationship between weight, height and dental caries among preschool children in China. PLoS One.

[3] Ayhan H, Suskan E, Yildirim S (1996). The effect of nursing or rampant caries on height, body weight and head circumference. J Clin Pediatr Dent.

[4] Acs G, Lodolini G, Kaminsky S, Cisneros GJ (1992). Effect of nursing caries on body weight in a pediatric population. Pediatr Dent.

[5] Sheiham A (2006). Dental caries affects body weight, growth and quality of life in pre-school children. Br Dent J.

[6] Al-Shahrani N, Al-Amri A, Hegazi F, Al-Rowis K, Al-Madani A, Hassan KS (2015). The prevalence of premature loss of primary teeth and its impact on malocclusion in the Eastern Province of Saudi Arabia. Acta Odontol Scand.

[7] Filstrup SL, Briskie D, da Fonseca M, Lawrence L, Wandera A, Inglehart MR (2003). Early childhood caries and quality of life: child and parent perspectives. Pediatr Dent.

[8] Pahel BT, Rozier RG, Slade GD (2007). Parental perceptions of children’s oral health: the Early Childhood Oral Health Impact Scale (ECOHIS). Health Qual Life Outcomes.

[9] Muirhead VE, Quayyum Z, Markey D (2018). Children’s toothache is becoming everybody’s business: where do parents go when their children have oral pain in London, England? A cross-sectional analysis. BMJ Open.

[10] Borges TS, Vargas-Ferreira F, Kramer PF, Feldens CA (2017). Impact of traumatic dental injuries on oral health-related quality of life of preschool children: a systematic review and meta-analysis. PLoS One.

[11] de Oliveira LJ, Torriani DD, Correa MB, Peres MA, Peres KG, Matijasevich A, Dos Santos IS, Barros AJ, Demarco FF, Tarquinio SB (2015). Oral mucosal lesions’ impact on oral health-related quality of life in preschool children. Community Dent Oral Epidemiol.

[12] Vieira-Andrade RG, Martins-Júnior PA, Corrêa-Faria P, Marques LS, Paiva SM, Ramos-Jorge ML (2015). Impact of oral mucosal conditions on oral health-related quality of life in preschool children: a hierarchical approach. Int J Paediatr Dent.

[13] Wong NH, Tran C, Pukallus M, Holcombe T, Seow WK (2012). A three-year retrospective study of emergency visits at an oral health clinic in south-East Queensland. Aust Dent J.

[14] Aaronson NK (1988). Quality of life: what is it? How should it be measured?. Oncology (Williston Park).

[15] Ruff RR, Sischo L, Chinn CH, Broder HL (2017). Development and validation of the child oral health impact profile - preschool version. Community Dent Health.

[16] Tsakos G, Blair YI, Yusuf H, Wright W, Watt RG, Macpherson LM (2012). Developing a new self-reported scale of oral health outcomes for 5-year-old children (SOHO-5). Health Qual Life Outcomes.

[17] Rebok G, Riley A, Forrest C, Starfield B, Green B, Robertson J (2001). Elementary school-aged children's reports of their health: a cognitive interviewing study. Qual Life Res.

[18] Hetherington EM, Parke RD, Locke VO (2003). Child psychology: a contemporary viewpoint.

[19] Bekes K, Omara M, Safar S, Stamm T (2019). The German version of Early Childhood Oral Health Impact Scale (ECOHIS-G): translation, reliability, and validity. Clin Oral Investig.

[20] Bordoni N, Ciaravino O, Zambrano O, Villena R, Beltran-Aguilar E, Squassi A (2012). Early Childhood Oral Health Impact Scale (ECOHIS). Translation and validation in Spanish language. Acta Odontol Latinoam.

[21] Farsi NJ, El-Housseiny AA, Farsi DJ, Farsi NM (2017). Validation of the Arabic version of the early childhood Oral health impact scale (ECOHIS). BMC Oral Health.

[22] Hashim AN, Yusof ZY, Esa R (2015). The Malay version of the Early Childhood Oral Health Impact Scale (Malay-ECOHIS-assessing validity and reliability). Health Qual Life Outcomes.

[23] Jabarifar SE, Golkari A, Ijadi MH, Jafarzadeh M, Khadem P (2010). Validation of a Farsi version of the Early Childhood Oral Health Impact Scale (F-ECOHIS). BMC Oral Health.

[24] Jankauskienė B, Narbutaitė J, Kubilius R, Gleiznys A (2012). Adaptation and validation of the early childhood oral health impact scale in Lithuania. Stomatologija..

[25] Lee GH, McGrath C, Yiu CK, King NM (2009). Translation and validation of a Chinese language version of the Early Childhood Oral Health Impact Scale (ECOHIS). Int J Paediatr Dent.

[26] Li S, Veronneau J, Allison PJ (2008). Validation of a French language version of the Early Childhood Oral Health Impact Scale (ECOHIS). Health Qual Life Outcomes.

[27] Martins-Júnior PA, Ramos-Jorge J, Paiva SM, Marques LS, Ramos-Jorge ML (2012). Validations of the Brazilian version of the Early Childhood Oral Health Impact Scale (ECOHIS). Cad Saude Publica.

[28] Nzomiwu CL, Sote EO, Oredugba FA (2018). Translation and validation of the Nigerian Pidgin English version of the Early Childhood Oral Health Impact Scale (NAIJA ECOHIS). West Afr J Med.

[29] Peker K, Uysal Ö, Bermek G (2011). Cross - cultural adaptation and preliminary validation of the Turkish version of the early childhood oral health impact scale among 5-6-year-old children. Health Qual Life Outcomes.

[30] Scarpelli AC, Oliveira BH, Tesch FC, Leão AT, Pordeus IA, Paiva SM (2011). Psychometric properties of the Brazilian version of the Early Childhood Oral Health Impact Scale (B-ECOHIS). BMC Oral Health.

[31] Randrianarivony J, Ravelomanantsoa J, Razanamihaja N (2020). Evaluation of the reliability and validity of the Early Childhood Oral Health Impact Scale (ECOHIS) questionnaire translated into Malagasy. Health Qual Life Outcomes.

[32] Bureau of Dental Health (2018). The 8th Thailand national oral health survey report 2017.

[33] Bureau of Dental Health (2013). The 7th Thailand national oral health survey report 2012.

[34] Nora ÂD, da Silva Rodrigues C, de Oliveira Rocha R, Soares FZM, Minatel Braga M, Lenzi TL (2018). Is caries associated with negative impact on oral health-related quality of life of pre-school children? A systematic review and meta-analysis. Pediatr Dent.

[35] Zaror C, Pardo Y, Espinoza-Espinoza G, Pont À, Muñoz-Millán P, Martínez-Zapata MJ (2019). Assessing oral health-related quality of life in children and adolescents: a systematic review and standardized comparison of available instruments. Clin Oral Investig.

[36] Guillemin F, Bombardier C, Beaton D (1993). Cross-cultural adaptation of health-related quality of life measures: literature review and proposed guidelines. J Clin Epidemiol.

[37] Wild D, Grove A, Martin M, Eremenco S, McElroy S, Verjee-Lorenz A (2005). Principles of good practice for the translation and cultural adaptation process for patient-reported outcomes (PRO) measures: report of the ISPOR task force for translation and cultural adaptation. Value Health.

[38] Bonett DG (2002). Sample size requirement for testing and estimating coefficient alpha. J Educ Behav Stat.

[39] Walter SD, Eliasziw M, Donner A (1998). Sample size and optimal designs for reliability studies. Stat Med.

[40] Bureau of Health Promotion (2005). Growth charts for Thai children 2005.

[41] Social Statistics Bureau (2011). Household income and expenditure in Bangkok 2011.

[42] Social Statistics Bureau (2019). The reading of population survey 2018.

[43] Tsakos G, Bernabé E, O'Brien K, Sheiham A, de Oliveira C (2008). Comparison of the self-administered and interviewer-administered modes of the child-OIDP. Health Qual Life Outcomes.

[44] Varni JW, Burwinkle TM, Lane MM (2005). Health-related quality of life measurement in pediatric clinical practice: an appraisal and precept for future research and application. Health Qual Life Outcomes.

[45] Gift HC, Atchison KA (1995). Oral health, health, and health-related quality of life. Med Care.

[46] Novaes TF, Pontes LRA, Freitas JG, Acosta CP, Andrade KCE, Guedes RS (2017). Responsiveness of the early childhood oral health impact scale (ECOHIS) is related to dental treatment complexity. Health Qual Life Outcomes.

[47] Wong HM, McGrath CPJ, King NM (2011). Rasch validation of the early childhood oral health impact scale. Community Dent Oral Epidemiol.

[48] Prieto L, Alonso J, Lamarca R (2003). Classical test theory versus Rasch analysis for quality of life questionnaire reduction. Health Qual Life Outcomes.

