# Correction to: Psychometric evaluation of the Thai version of the Early Childhood Oral Health Impact Scale (Th-ECOHIS): a cross sectional validation study

**DOI:** 10.1186/s12903-021-01483-6

**Published:** 2021-03-26

**Authors:** Pattarawadee Leelataweewud, Varangkanar Jirarattanasopha, Chantana Ungchusak, Warangkana Vejvithee

**Affiliations:** 1 Department of Pediatric Dentistry, Faculty of Dentistry, Mahidol University, No. 6, Yothi Road, Ratchathewi District, Bangkok, 10400 Thailand; 2 Bureau of Dental Health, Department of Health, Ministry of Public Health, No. 88/22, Tiwanond Road, Nonthaburi, 11000 Thailand

## Correction to: BMC Oral Health (2021) 21:64 10.1186/s12903-020-01332-y

After publication of the original article [1], the authors identified an error in the Results section: the below text is missing and it should be placed before the Discussion:

Overall, 55.1% of caregivers reported that at least one aspect related to child’s oral health had affected their children and family (Table 3). The percentage of proxy respondents who reported oral health problems affecting their family (46.3%) was higher than the percentage of those who reported the problems affecting their children (36.9%). The three most prevalent responses in the child impact section was “pain in the teeth, mouth, or jaws” (26.7%), followed by “became irritable or frustrated” (25.7%) and “difficulty in eating some foods” (16.8%) (Table 2). In the family impact section, the two most frequently reported impacts were “parents or family members feeling guilty” (39.7%) and “being upset” (39.3%) (Table 2).

## Reliability

The overall reliability of the Th-ECOHIS showed good results (Table 4). The mean inter-item correlations (Cronbach’s alpha coefficient) of the total ECOHIS items, child impact section, and family impact section were 0.85, 0.84, and 0.71, respectively. The test–retest reliability scores (intraclass correlation coefficient, ICC) of the Th-ECOHIS were 0.87 for the total of the items, 0.78 for the child impact section, and 0.87 for the family impact section.

## Validity

The convergent validity was analyzed using the Spearman correlation coefficient, which showed a moderate correlation for the global oral health rating and total Th-ECOHIS score (*r* = 0.604; *p* < 0.01) (Table 5). The discriminant validity was assessed by comparing the Th-ECOHIS scores for the severity of caries experience and dental treatment need. Variations were apparent in the ECOHIS and the two subscale scores (child impact and family impact sections) (*p* < 0.001) for different caries status (Table 6). Children with caries had higher ECOHIS scores than caries-free children. Children with severe ECC had significantly higher Th-ECOHIS scores than children with ECC did. Differences of the Th-ECOHIS were significant among treatment need categories (*p* < 0.001). Children who had dental treatment need had a higher Th-ECOHIS score than those who did not (Table 6).

Furthermore, Tables 4, 5 and 6 are missing. The tables are given below:

**Table 4:** Reliability analyses of the Th-ECOHIS: internal consistency and test-retest reliability

Impact	Internal consistency Cronbach’s alpha	Test- retest reliability [ICC] (95% CI)]
Child impact section	.842	0.78 (0.40, 0.92)
Family impact section	.706	0.87 (0.64, 0.95)
All sections	.854	0.87 (0.63, 0.95)

Intraclass correlation coefficient (two-way mixed effects model)

**Table 5:** Convergent validity of the Th-ECOHIS

	Child impact	Family impact	Total score
Global oral health rating	0.423*	0.622*	0.604*

*p <  0.01

**Table 6:** Discriminant validity of the Th-ECOHIS

Caries experience	Caries-free (*n* = 130)	dmft 1–3 (*n* = 47)	dmft ≥4 (*n* = 37)	*p*-value
Child impact section				
Mean(SD)	1.30 (3.68)	2.47 (3.43)	3.97 (4.69)	
Median(IQR)	0 (0)	1 (4)	3 (7)	< 0.001
Family impact section				
Mean(SD)	1.52 (2.59)	2.45 (2.51)	3.68 (3.16)	
Median(IQR)	0 (2)	2 (4)	4 (6)	< 0.001
All sections				
Mean(SD)	2.51 (4.13)	4.74 (4.83)	7.65 (7.05)	
Median(IQR)	0 (4)	4 (8)	7 (11)	< 0.001

Treatment need	No treatment (*n* = 130)	Need filling (*n* = 65)	Need pulp treatment / extraction (*n* = 19)	*p*-value
Child impact section				
Mean(SD)	1.18 (3.47)	3.29 (4.51)	3.68 (3.38)	
Median(IQR)	0 (0)	1 (6)	3 (7)	< 0.001
Family impact section				
Mean(SD)	1.45 (2.60)	3.05 (2.96)	3.32 (2.11)	
Median (IQR)	0 (2)	3 (6)	4 (3)	< 0.001
All sections				
Mean(SD)	2.64 (5.25)	6.34 (6.78)	7.00 (4.49)	
Median(IQR)	0 (4)	5 (12)	8 (7)	< 0.001

The original article has been corrected.
